# Association between subjective degree of influence in class and thinness among adolescents in Japan

**DOI:** 10.3389/fped.2022.938139

**Published:** 2023-01-09

**Authors:** Nanako Ishikawa, Yuna Koyama, Satomi Doi, Aya Isumi, Takeo Fujiwara

**Affiliations:** ^1^ Department of Global Health Promotion, Tokyo Medical and Dental University, Tokyo, Japan; ^2^ Japan Society for the Promotion of Science (JSPS), Tokyo, Japan

**Keywords:** degree of influence, thinness, social comparison, adolescence, school

## Abstract

Social status in school, measured by subjective degree of influence in class (DOI), may influence thinness among adolescents. This study examined the association between subjective degree of influence in class and thinness among Japanese adolescents. Data were obtained from the Kochi Child Health Impact of Living Difficulty (K-CHILD) study in 2016, which Was a population-based study targeting 5th, 8th and 11th grade adolescents living in Kochi Prefecture, Japan (*N* = 9,998). DOI was assessed by adolescents *via* questionnaire. Weight and height were given by caregivers for 5th grade adolescents, whilst they were self-reported for 8th and 11th grade adolescents. Collected data on weight and height were used to calculate body mass index z-scores of WHO standards. Models included grade, gender, number of friends, household income, location of school and depressive symptoms as covariates. The results showed that both high and low DOI were positively associated with thinness after adjustment for other individual covariates (high DOI, OR = 1.59, 95% CI 1.05–2.43; low DOI, OR = 2.04, 95% CI 1.36–3.06). Further stratification by gender revealed that low DOI was positively associated with thinness (OR = 2.14, 95% CI 1.34–3.44) among boys, but there was no association among girls. Both high and low DOI were associated with the risk of being thin in adolescents. Focusing on DOI for adolescents may be important to address thinness among adolescents. Further studies are needed to examine the causality between DOI and thinness in adolescents.

## Introduction

Thinness in adolescence can affect health in many aspects; it can affect individual's physical health (1) and mental health, including eating disorder (2–5), which increases the risk of anxiety disorders and depressive disorders (1). Thinness could also affect the health of the future generation through various mechanisms; one of which is small-for-gestational age (SGA) (6). That is, low maternal pregravid BMI increases the incidence of SGA (6, 7), and SGA is associated with childhood poor neurological development including poor school performance as well as adulthood physical and psychological health (6, 8–10). Although further studies are required, paternal thinness may also affect SGA (11, 12). Thus, thinness in adolescence is not only a problem for adolescents themselves but also for the future generation.

To prevent thinness, it is needed to elucidate its determinants. Well-known determinants of thinness include socioeconomic status and subjective social status. For example, one study has shown a positive association between high subjective social status and thinness among South Korean women (13). Other studies in developed countries such as England (14) and Scotland (14, 15) reported that children living in more deprived areas were more likely to be thin than those living in the least deprived areas. Furthermore, number of studies in developing countries showed association between low socioeconomic status and thinness (14, 16).

However, there is no previous study exploring the association between thinness and social status unique to adolescents in developed countries. We propose the use of subjective degree of influence in class (DOI), an indicator of social status within school classroom, to determine its relationship with thinness in adolescence. DOI can measure social status of adolescents, which is largely influenced by social comparison between peers at school. According to social comparison theory, social comparison is the tendency to compare oneself to others to understand where and how one fits in society and it can possibly cause changes in individuals (17–19). As peers can be extremely important targets of social comparison for adolescents (19, 20), peers shape many behaviors and cognitions of adolescents, including setting norms and expectations related to appearance concerns (19, 21).

We hypothesize that both low and high DOI would be associated with thinness for girls and muscularity for boys, albeit with different mechanisms. Adolescents with low DOI may have a higher risk of being thin, compared to those with moderate DOI. This is because women tend to evaluate their appearance against women who they perceive to be superior to themselves (18), (i.e., peers with higher DOI) and past studies reported that common ideal regarding appearance is thinness for women and muscularity for men (22, 23). From these findings, social comparison targets of adolescents with low DOI are likely to be peers who are thinner or muscular than themselves, and they may take extreme measures to lose weight, eventually becoming thin.

Adolescents with high DOI, on the other hand, could compare their appearances to peers in the same social groups, such as within friendship groups (i.e., peers with same DOI). Previous study suggested that in high-income countries, school friends tend to have similar Body Mass Index (BMI), and an individual belonging to a friendship group with a high frequency of reported dieting and weight loss reported the following more frequently on average: dieting and peer pressure to lose weight to become thin (24). Additionally, girls are more prone to peers' dieting and weight control behaviors (25). Therefore, girls in the high DOI group could be pressured heavily to be thin through comparison with other slim members of the same DOI group as well as to maintain peer likeability (26). For boys with high DOI, although there are scarce literature on boys' thinness and body image (26), a preceding study revealed that among boys there were significant associations between friends' muscle-enhancing behaviors (25). Thus, we hypothesize that girls with high DOI will have a higher prevalence of being thin, and boys with high DOI will gain muscle whilst losing fat, resulting in a higher prevalence of being thin compared to those with moderate DOI.

To summarize, we hypothesize that adolescents with higher or lower DOI than average will have a higher risk of being thin. It is important to elucidate the association between DOI and thinness in adolescents for the following reasons. First, DOI is a measure that can be easily measured by adolescents themselves (21), allowing them to monitor the risk of thinness by themselves. Second, if DOI is modifiable through internvention at school, it will be possible to prevent thinness among adolescents. Third, DOI is a measure applicable to children from elementary to high school. Thus, it allows an early intervention of thinness at the start of puberty, minimizing the negative effects of thinness on development.

To investigate the association between DOI and thinness, Japan is one of the most appropriate countries, because, among the OECD countries, its proportion of thinness is one of the highest (5, 6), which results in the second-highest prevalence of low-birth-weight infants (27, 28). In addition, self-esteem of adolescents is low, possibly due to high peer pressure (29), which is strongly related with DOI. Thus, this study aimed to examine the association between DOI and thinness amongst adolescents, for both boys and girls, in Japan.

## Materials and methods

### Study designs and subjects

**Figure 1:**
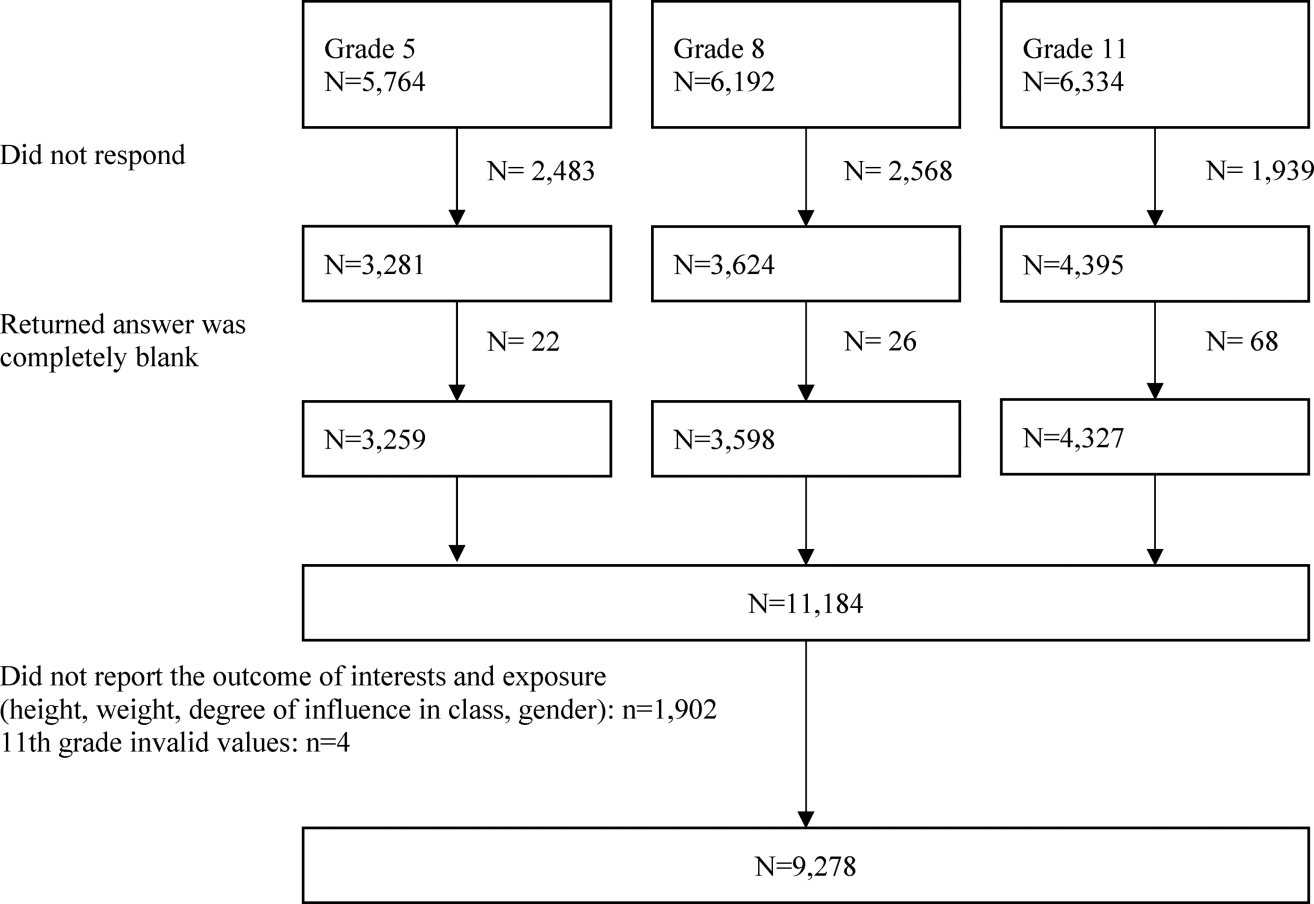
Requirement flowchart.

We used cross-sectional data collected in 2016 in the Kochi Child Health Impact of Living Difficulty (K-CHILD) study, which was a population-based study established to evaluate the determinants of health among children and their caregivers in Kochi Prefecture, Japan (30). Self-report questionnaires were distributed to adolescents enrolled in 5th, 8th and 11th grade in Kochi prefecture except for those enrolled in correspondence high schools and special needs schools. In total, 18,290 adolescents received the questionnaires. In Kochi City, the questionnaires were returned *via* mail, and outside of Kochi City, they were collected and returned *via* school. A total of 11,200 returned the questionnaires (participation rate: 61.2%) and 11,184 of them contained a valid response (valid response rate: 61.1%). Among these valid responses, 1,902 responses were excluded as the outcome of interests and exposure (i.e., data on thinness and DOI) were missing, and 4 responses of adolescents in 11th grade with invalid birth year values were excluded, resulting in the analytic sample size of 9,278 (see Figure 1).

## Measurements

### Thinness

Weight and height were reported by caregivers for 5th grade adolescents, whilst they were self-reported for 8th and 11th grade adolescents. BMI was calculated according to the WHO Child Growth Standards and was expressed as z-scores, representing the deviations in standard deviation units from the mean of a standard normal distribution of BMI specific to age and sex (31, 32). Following the cut-off point recommended by the WHO, BMI-for-age z-score < −2SD was defined as thinness among adolescents (33, 34). To see the components of BMI, height was also used as a secondary outcome, converting z-scores specific to age and sex according to the WHO Child Growth Standards (33). For analysis, as we are interested in thinness, i.e., taller height, height-for-age z-score >=1SD was defined as tallness among adolescents.

### Degree of influence (DOI)

DOI was assessed by adolescents *via* a questionnaire, which was used in the previous study (30). We asked the question “How influential are your opinions and behaviors on your classmates?” and the answer choices were “1 = not at all”, “2 = a little”, “3 = to some extent”, “4 = very much”. For analysis, we categorized them into three divisions: high, moderate, and low. In the current study, “4 = very much” was categorized as high, “3 = to some extent” and “2 = a little” as moderate, and “1 = not at all” as low.

### Covariates

Covariates in the models included grade (5th, 8th, 11th), gender (boy, girl), number of friends one can share their worries with, household income, location of school (within Kochi City, outside Kochi City), and depressive symptoms. The rationale of these covariates is as follows. First, a previous study showed that the distribution of DOI was different for each grade (30), and in a different study conducted in Japan the prevalence of thinness differed with age in adolescents (35). Second, there was a difference in DOI distribution between boys and girls (30), and women tend to have a drive for thinness whilst men have a greater drive for muscularity, suggesting that pathways leading to thinness vary by gender (22, 23). Third, self-reported number of friends is positively associated with DOI (30), and weight status was also associated with the number of confidants in adolescents (36). Fourth, although inconsistent, previous studies reported that thinness is associated with household income (37–39). Household income also impacts one's perceived social rank (40), which is one's perceptions of the degree to which one feels inferior to others and looked down on (41). The link between household income and social rank may be weak in adolescents (30); nonetheless, awareness of one's economic status in class could influence DOI especially for adolescents of older age. Fifth, DOI distribution differed by location of school in the previous study (30), and many studies have shown that distribution of body weight in adolescents was associated with urban/rural difference (42). Lastly, females with thinness were more likely to show depressive symptoms (43), and adolescents with low self-reported social status were at higher risk of depression compared to those reporting medium or high social status (44). Thus, depression can be a confounder in this study. To assess depressive symptoms, we used the Japanese version of the Depression Self-Rating Scale (DSRS), modified from the English version (45). The total score ranged from 0 to 30, and a higher score indicated more severe depressive symptoms.

### Statistical analyses

To examine the association between DOI and thinness among adolescents, multiple logistic regression analyses were used. For Model 1, the following covariates were included: grade, gender, number of friends, household income, and location of the school. In addition to covariates in Model 1, depressive symptoms were added in Model 2. Further stratification by gender was conducted for both analyses on the associations between DOI and thinness and DOI and height, as these associations may differ by gender due to the following reasons. Women tend to have a drive for thinness, whilst men tend to have a drive for muscularity (22, 23). Analyses were performed with STATA SE statistical package, version 15 (StataCorp LP, College Station, TX, USA).

## Result

Demographic characteristics of the analytical sample are shown in Table 1. The percentages of boys and girls were similar (49.9% to 50.2%). More than half of the households reported a household income of JPY3.00–8.99 million (USD1= JPY110, as of Oct 2016). The percentage of children attending schools within Kochi City, the capital city of Kochi prefecture, and those attending schools outside Kochi City were 47.5% and 52.6%, respectively. More than half (58.5%) of the children reported that the number of friends one can share their worries with was above 3. Thinness was observed in 290 (3.1%) of the sample (boys 185 (2.0%), girls 105 (1.1%)). Around one-tenth reported that their DOI was “high” or “low”. There were no major differences in the distribution of DOI for boys and girls. DOI progressively decreased with age; in the high DOI group, proportion of 5th grade children was the highest (41.6%), and 11th grade children was the lowest (28.5%). On the other hand, proportion of low DOI group was the highest in the 11th grade children (49.9%). Furthermore, urban/rural difference in DOI distribution was observed. High DOI was more likely found among children outside Kochi City (61.5%) compared to children in Kochi City (38.5%). High DOI children had larger number of friends, that is, 50.6% of the high DOI group reported that they have more than 5 friends one can share their worries with, which was higher than moderate DOI group (38.5%) and low DOI group (15.8%). Moreover, depressive symptoms were higher among children in low DOI group compared to moderate DOI group, and moderate DOI children showed higher depressive symptoms than high DOI group.

**Table 1:** Sample characteristics (*N* = 9,278).

Variables		Degree of influence						Total (*N* = 9,278)	
		High (*N* = 878) 9.5%		Moderate (*N* = 7,563) 81.5%		Low (*N* = 837) 9.0%			
		*N* or Mean	% or SD	*N* or Mean	% or SD	*N* or Mean	% or SD	*N* or Mean	% or SD
School Grade	5th	365	41.6	2,155	28.5	152	18.2	2,672	28.8
	8th	263	30.0	2,476	32.7	267	31.9	3,006	32.4
	11th	250	28.5	2,932	38.8	418	49.9	3,600	38.8
Gender	Boy	496	56.5	3,706	49.0	423	50.5	4,625	49.9
	Girl	382	43.5	3,857	51.0	414	49.5	4,653	50.2
Household Income	0 JPY	13	1.5	105	1.4	19	2.3	137	1.5
	1-2.99M JPY	146	16.6	1,311	17.3	189	22.6	1,646	17.7
	3.00–5.99 M JPY	256	29.2	2,402	31.8	252	30.1	2,910	31.4
	6.00–8.99 M JPY	213	24.3	1,558	20.6	134	16.0	1,905	20.5
	≧9.00 M JPY	88	10.0	767	10.1	69	8.2	924	10.0
	Unknown/missing	162	18.5	1,420	18.8	174	20.8	1,756	18.9
Location of School	Within Kochi City	338	38.5	3,594	47.5	470	56.2	4,402	47.5
	Outside Kochi City	540	61.5	3,969	52.5	367	43.9	4,876	52.6
No. of friends you can share your worries with	0	63	7.2	813	10.8	283	33.8	1,159	12.5
	1–2	172	19.6	1,896	25.1	255	30.5	2,323	25.0
	3–4	156	17.8	1,642	21.7	140	16.7	1,938	20.9
	≧5	444	50.6	2,915	38.5	132	15.8	3,491	37.6
	Missing	43	4.9	297	3.9	27	3.2	367	4.0
Depressive symptoms (0-30)		6.57	5.2	9.37	5.2	14.03	6.1	9.53	5.5
Thinness^a^	+	38	4.3	202	2.7	50	6.0	290	3.1
	−	840	95.7	7,361	97.3	787	94.0	8,988	96.9
Tallness^b^	+	70	8.0	345	4.6	33	3.9	448	4.8
	−	808	92.0	7,217	95.4	804	96.1	8,829	95.2

^a^ Thinness was defined as BMI-for-age z-score WHO Growth reference 5–19 years < −2SD.

^b^ Tallness was defined as height-for-age z-score WHO Growth reference 5–19 years >= 1SD.

Abbreviation: JPY, Japanese Yen.

Table 2 shows the association between DOI and thinness among all children and stratified by gender. Interestingly, we found a U-shaped association between DOI and thinness; both children with high and low DOI showed significant association with thinness (high DOI, odds ratio (OR) = 1.65, 95% confidence interval (CI) 1.16–2.35; low DOI, OR = 2.32, 95% CI 1.68–3.18) in comparison with children with moderate DOI in the crude model. After covariate adjustment, both high and low DOI remained significantly associated with thinness (high DOI, OR = 1.59, 95% CI 1.05–2.43; low DOI, OR = 2.04, 95% CI 1.36–3.06). As for gender stratification, we found different associations between DOI and thinness; among boys, low DOI was positively associated with thinness (OR = 2.13, 95% CI 1.34–3.43), while high DOI did not show significant association with thinness (OR = 1.55, 95% CI 0.89–2.70). Among girls, high and low DOI were not statistically significantly associated with thinness in adjusted model (high DOI, OR = 1.63, 95% CI 0.85–3.13; low DOI, OR = 1.58, 95% CI 0.69–3.64).

**Table 2:** The association between degree of influence and thinness.

		Thinness^a^					
		Crude		Model 1		Model 2	
		OR	95% CI	OR	95% CI	OR	95% CI
Total (*N* = 9,278)							
Degree of influence	High	**1.65**	(1.16–2.35)	**1**.**57**	(1.03–2.38)	**1**.**59**	(1.05–2.43)
	Moderate	ref.		ref.		ref.	
	Low	**2.32**	(1.68–3.18)	**2**.**12**	(1.44–3.13)	**2**.**04**	(1.35–3.06)
Boy (*N* = 4,625)							
Degree of influence	High	1.43	(0.91–2.25)	1.45	(0.84–2.51)	1.55	(0.89–2.70)
	Moderate	ref.		ref.		ref.	
	Low	**3.07**	(2.11–4.45)	**2**.**36**	(1.50–3.71)	**2**.**13**	(1.34–3.43)
Girl (*N* = 4,653)							
Degree of influence	High	**1.93**	(1.10–3.38)	1.73	(0.91–3.29)	1.63	(0.85–3.13)
	Moderate	ref.		ref.		ref.	
	Low	1.17	(0.60–2.27)	1.42	(0.63–3.20)	1.58	(0.69–3.64)

Bold indicates *p* < 0.05.

^a^ Thinness was defined as BMI-for-age z-score WHO Growth reference 5–19 years < −2SD.

Model 1 adjusted for grade, gender, no. of friends, income, location of school.

Model 2 adjusted for depressive symptoms in addition to Model 1.

Table 3 shows the association between DOI and height. High DOI was positively associated with greater height (OR = 1.55, 95% CI 1.14–2.12), whilst low DOI was not associated with greater height (OR = 0.89, 95% CI 0.57–1.40). High DOI was positively associated with greater height for boys (OR = 1.94, 95% CI 1.42–2.66). No significant association was seen between DOI and greater height for girls (high DOI, OR = 1.05, 95% CI 0.60–1.86; low DOI, OR = 0.97, 95% CI 0.47–2.00).

**Table 3:** The association between degree of influence and tallness.

		Tallness^a^					
		Crude		Model 1		Model 2	
		OR	95% CI	OR	95% CI	OR	95% CI
Total (*N* = 9,278)							
Degree of influence	High	**1.80**	(1.37–0.59)	**1**.**61**	(1.19–2.18)	**1**.**55**	(1.14–2.12)
	Moderate	ref.		ref.		ref.	
	Low	0.85	(0.59–1.22)	0.92	(0.60–1.43)	0.89	(0.57–1.40)
Boy (*N* = 4,625)							
Degree of influence	High	**1.94**	(1.42–2.66)	**1**.**90**	(1.31–2.73)	**1**.**83**	(1.25–2.68)
	Moderate	ref.		ref.		ref.	
	Low	0.95	(0.62–1.47)	0.86	(0.49–1.51)	0.78	(0.43–1.40)
Girl (*N* = 4,653)							
Degree of influence	High	1.24	(0.73–2.09)	1.04	(0.59–1.82)	1.05	(0.60–1.86)
	Moderate	ref.		ref.		ref.	
	Low	0.66	(0.35–1.27)	0.97	(0.48–1.97)	0.97	(0.47–2.00)

Bold indicates *p* < 0.05.

^a^ Tallness was defined as height-for-age z-score WHO Growth reference 5–19 years >= 1SD.

Model 1 adjusted for grade, gender, no. of friends, income, location of school.

Model 2 adjusted for depressive symptoms in addition to Model 1.

## Discussion

In the current study, adolescents with both high and low DOI were more likely to be thin compared to moderate DOI. Furthermore, when stratified by gender, low DOI showed a significant positive association with thinness among boys. In addition, high DOI showed a positive association with greater height, and when stratified by gender, high DOI showed a positive association with greater height among boys, whilst no association was seen among girls.

Our findings on the positive association between high DOI and thinness accorded with the previous studies examining associations between social status and thinness in adults, such as the positive association between high subjective social status and thinness among South Korean women (13).

However, we could not deny the existence of unmeasured confounders, such as personality traits unique to high DOI children. A previous study has shown that in early adolescents, having energy and openness, both measured by the Big Five Personality Test, positively influence one's perceived social acceptance (46). In other words, early adolescents who are social and have high intellectual curiosity are likely to regard themselves as being socially accepted by their schoolmates. Due to these traits, children with high DOI could gather information related to weight loss and beauty standards from media and friends. Frequent exposure to such topics along with their high intellectual curiosity may result in them attributing their popularity to their physical appearances, i.e., being thin, as extraversion is related to thin-ideal internalization (47). Thus, they are likely to lose weight to maintain their social position. Furthermore, there could be reverse causation. As friendship groups tend to mimic weight-related behaviors (25), comparing one's weight to the thin group members will induce thinness in those who were not thin before.

The positive association between low DOI and thinness was also in line with the previous findings, such as the positive association between low economic status and thinness among children in England (14) and Scotland (14, 15), and can be explained by low self-esteem and psychological distress. Low DOI children may have low self-esteem, and it is known that compared to peers with high self-esteem, those with low self-esteem perceive more pressure from the media and peers regarding physical appearances, such as losing weight (48). However, these children will likely remain in the low DOI group even after losing weight, as their social standing in the classroom is already set. Similarly, psychological distress from being in the low DOI group could lead to thinness. Those with low social rank will have mental health problems such as depression and social anxiety (25, 44), and a recent study has revealed that chemogenetic inhibition of the same neurons induces anxiety, depression, and reduced feeding (49). Furthermore, neglect is a possible confounder which was unexplored in this study. Prior studies have shown that child neglect resulted in failure to thrive in infants and children (50). It is also reported that children who were victims of neglect reported lower self-esteem (51), limited peer interactions (51, 52), and unpopularity from peers (51, 53), which may lead to low DOI. Although current study to examine the association between DOI and thinness, an adolescent-unique measurement of social status, and thinness in adolescents, future studies including further assessments of potential confounders as well as longitudinal design were warranted.

We also found that the positive association between low DOI and thinness for boys remained significant when stratified by gender, whilst no association was seen between thinness and the following: high DOI for boys, high DOI for girls, and low DOI for girls. The positive association between low DOI and thinness for boys was in accordance with our hypothesis that boys with low DOI were driven to extreme musclebuilding behavior, which resulted in thinness. We noted the potential reverse causation related to the sociocultural ideal body of boys, known as a V-shaped muscular build (26, 54, 55). That is, boys who are thin and not muscular, could report low DOI as they do not meet the sociocultural ideal. No statistically significant association between high or low DOI and thinness for girls may be an indication that social status may not be heavily influenced by body type for girls. However, as point estimates of the other associations were similar to all children samples, small sample size matters for non-significant associations. In addition, high DOI was positively associated with greater height for boys. This result was supported by past studies reporting that men who were taller often took on leadership roles within groups (56), and were often perceived as competent and talented by others (49, 56). Considering that there was no association between high DOI and thinness for boys, this result indicates boys may have a greater drive to be tall than to be thin.

There are several limitations to this study. First, due to a cross-sectional study, the causality of DOI and thinness could not be examined. Further longitudinal study is warranted to evaluate this causality. Second, the questionnaire was self-reported; measures such as weight and height used to calculate BMI were either reported by the parents or children themselves. However, the validity of self-reported BMI values of children has been proven previously (57). Third, there was no measurement of personality, such as the use of Big 5 personality test, needed to understand the pathway of DOI and thinness.

Despite these limitations, this is the first study to report the importance of the subjective social rank of children on their health using DOI, with both high and low levels of DOI positively associated with thinness among adolescents, suggesting that both high and low levels of DOI could be a precursor of thinness for adolescents. This study has several key implications. First, DOI could be a measure of social rank within classroom for adolescents, as the result of this study is consistent with previous studies which reported positive association between high subjective social status among adults and thinness (13), as well as low socioeconomic status among children and thinness (14, 15). Therefore, further investigation of the causal association between DOI and thinness can be conducted. Second, because DOI can be easily measured by adolescents themselves (21), measuring DOI will lead to prompt intervention and evaluation of the risk population. Lastly, it will be possible to prevent thinness among adolescents on a wide scale using DOI, a measure applicable to teenagers. DOI is a concept that is understandable for elementary school children, allowing prevention of thinness before the growth spurt. Furthermore, DOI will be an important measure not only for Japan but also for other countries, where students spend majority of their school life with their classmates.

In conclusion, both high and low DOI were associated with the risk of being thin in adolescents, which is one of the important risk factors of eating disorders. Focusing on DOI for adolescents may be important to address thinness among adolescents, and further studies are needed to examine the causality between DOI and thinness in adolescents.

## Data Availability

The datasets presented in this article are not readily available because it is the part of population-based study conducted by the corresponding author. However, upon a reasonable request, the datasets would be available from the corresponding author. Requests to access the datasets should be directed to TF, fujiwara.hlth@tmd.ac.jp.
